# Nephroprotective Activity of Papaloquelite (*Porophyllum ruderale*) in Thioacetamide-Induced Injury Model

**DOI:** 10.3390/plants11243460

**Published:** 2022-12-10

**Authors:** María José Vázquez-Atanacio, Mirandeli Bautista, Manasés González-Cortazar, Antonio Romero-Estrada, Minarda De la O-Arciniega, Araceli Castañeda-Ovando, Carolina G. Sosa-Gutiérrez, Deyanira Ojeda-Ramírez

**Affiliations:** 1 Área Académica de Medicina Veterinaria y Zootecnia, Instituto de Ciencias Agropecuarias, Universidad Autónoma del Estado de Hidalgo, Av. Universidad Km 1, Ex-Hda. de Aquetzalpa, Tulancingo 43600, Hidalgo, Mexico; 2 Área Académica de Farmacia, Instituto de Ciencias de la Salud, Universidad Autónoma del Estado de Hidalgo, Ex Hacienda la Concepción s/n, San Agustín Tlaxiaca 42160, Hidalgo, Mexico; 3 Centro de Investigación Biomédica del Sur, Instituto Mexicano del Seguro Social, Argentina No. 1., Centro, Xochitepec 62790, Morelos, Mexico; 4 Departamento de Madera, Celulosa y Papel, Centro Universitario de Ciencias Exactas e Ingenierías, Universidad de Guadalajara, Km 15.5 Carretera Guadalajara-Nogales, Col. Las Agujas, Zapopan 45100, Jalisco, Mexico; 5 Área Académica de Química, Instituto de Ciencias Básicas e Ingeniería, Universidad Autónoma del Estado de Hidalgo, Pachuca-Tulancingo km 4.5 Carboneras, Mineral de la Reforma 42184, Hidalgo, Mexico

**Keywords:** *Porophyllum ruderale*, antioxidant, anti-inflammatory, nephroprotective

## Abstract

Acute kidney injury and impaired kidney function is associated with reduced survival and increased morbidity. *Porophyllum ruderale* is an edible plant endemic to Mexico used in Mexican traditional medicine. The aim of this study was to evaluate the nephroprotective effect of a hydroalcoholic extract (MeOH:water 70:30, *v/v*) from the aerial parts of *P. ruderale* (HEPr). Firstly, in vitro the antioxidant and anti-inflammatory activity of HEPr was determined; after the in vivo nephroprotective activity of HEPr was evaluated using a thioacetamide-induced injury model in rats. HEPr showed a slight effect on LPS-NO production in macrophages (15% INO at 40 µg/mL) and high antioxidant activity in the ferric reducing antioxidant power (FRAP) test, followed by the activity on DPPH and ABTS radicals test (69.04, 63.06 and 32.96% of inhibition, respectively). In addition, values of kidney injury biomarkers in urine (urobilinogen, hemoglobin, bilirubin, ketones, glucose, protein, pH, nitrites, leukocytes, specific gravity, and the microalbumin/creatinine) and serum (creatinine, urea, and urea nitrogen) of rats treated with HEPr were maintained in normal ranges. Finally, 5-*O*-caffeoylquinic, 4-*O*-caffeoylquinic and ferulic acids; as well as 3-*O*-quercetin glucoside and 3-*O*-kaempferol glucoside were identified by HPLC as major components of HEPr. In conclusion, *Porophyllum ruderale* constitutes a source of compounds for the treatment of acute kidney injury.

## 1. Introduction

Acute kidney injury (AKI) is a clinical syndrome characterized by an abrupt or rapid (hours to days) decline in renal filtration function, with the accumulation of products of nitrogen metabolism such as creatinine and urea and other clinically unmeasured waste products [1]; additionally, renal-tubular injury, inflammation and vascular dysfunction are observed [2]. AKI has become a global health problem and it is generally associated with a high ratio of mortality, mainly in developing countries and has an independent effect on the risk of death [3]. In addition, the treatment of this illness represents high costs for the health system of any country [4].

The main cause of AKI is non-controlled inflammation [5]. Inflammation is a complex integrated response designed to eliminate any noxious stimuli introduced into the host from the internal and external environment [6]. A normal inflammatory response is characterized by the infiltration of leukocytes and the release of other activated inflammatory mediators at the site of injury/infection that will eventually resolve or regulate with the release of these mediators [7]. It is important to mention that within the cells that participate in the inflammatory process there are two types: those that are permanently found in the tissues (mast cells and endothelial cells) and those that can migrate and access the affected site from the blood (polymorphonuclear neutrophils, monocytes, macrophages, and lymphocytes) [8]. These cells produce many active molecules that are direct or indirect mediators of the inflammatory process, including nitric oxide (NO), which is responsible for the regulation of numerous physiological processes, such as neurotransmission, smooth muscle contractility, platelet reactivity and cytotoxic activity of immune cells [9].

Following the beginning of the inflammation process, immune cell chemotaxis and infiltration, production of reactive oxygen species and cell-derived mediators create an intense inflammatory reaction that potentiates renal injury [5], for that reason the control of oxidative stress is another pivotal process in AKI development. Mainly the renal mitochondria are affected by the accelerated production of superoxide anion, hydrogen peroxide and hydroxyl radicals; which significantly increases the serum levels of the main renal markers such as creatinine and urea nitrogen [9,10].

Due to the importance of the problem, the pharmacological activities of different natural compounds have been studied to help in renal protection against nephrotoxicity caused by different compounds such as CCl_4_ and thioacetamide (TAA) [11,12]. Thioacetamide is an organosulfur compound used as a fungicide and in the production of stabilizers, catalyst, electroplating additives, polymerization inhibitors, denying aids, mineral processing agents and photograph development chemicals [13,14]. Despite several uses for TAA, this compound is an important toxin due to the generation of toxic fumes that can be inhaled, ingested, or absorbed through the skin [15]. TAA can affect organs depending on the exposition time; for example, a single dose administration produced centrilobular hepatic necrosis and nephrotoxic damage; prolonged exposure produces bile duct proliferation and liver cirrhosis [16]. Furthermore, TAA can produce inflammation, because it downregulates the expression of interleukin-1β (IL-1B) and TNF-a genes and upregulates the expression of interferon-γ (IFN-g) and interleukin-8 (IL-8) genes [14].

The Porophyllum genus, belonging to the Asteraceae family, comprises 25 species scattered among the United States, Mexico, Central America and South America. Seventeen of these species are found in Mexico and six inhabit the central-western region of Argentina. They are annual or perennial plants that present intense green leaves with numerous aromatic glands and have a strong flavor. The most widely distributed species of this genus in Mexico is *Porophyllum ruderale*, which is known by the names “papalo” or “papaloquelite”, a name derived from the Nahuatl “Papaloquílitl”, where “pápalotl” means butterfly, and “quilitl” means quelite [17,18,19].

*Porophyllum ruderale* is an annual edible herb with opposite and alternate leaves, with petioles 6–25 mm long; elliptical or oval and wavy on the margin. Its flowers are numerous, hermaphrodite, with a greenish or purplish corolla, tubular with the presence of long, thin, and curved branches. *P. ruderale* is a phytogenetic resource of great importance for food and agriculture, it has been consumed in México since pre-Hispanic times [18], and nowadays is consumed either alone or in combination due to its organoleptic and nutritionally properties [19]; in addition, due to its adaptation to environmental conditions, it can be consumed throughout the year [20]. Furthermore, *P. ruderalle* is used in the perfume and pesticide industry, due to the large quantities of strong-smelling volatile essential oils [18,19,20,21,22,23].

Medicinally, in México papaloquelite has been used in infusion and topically as a poultice for the treatment of several illnesses. For instance, in Tabasco and Oaxaca states, papalo is used as a local analgesic for toothache, headache and earache through the external application of its leaves on the affected part. In addition, an infusion of stems and leaves is used for the treatment of stomach pain, ulcers, vomiting, hemorrhoids, dysentery, colic and indigestion. In Yucatán state, a poultice of leaves of *P. ruderalle* is used to treat skin problems; while in Michoacán and Veracruz states an infusion of the root or leaves is used as a laxative, emmenagogue and for the treatment of liver diseases and hypertension [18,19]. Furthermore, *P. ruderalle* is used in folk medicine in Brazil for leishmaniasis, closing wounds, general pain, and internal bruising [24].

Some pharmacological properties have also been described for this plant, such as antioxidant, antimicrobial, anti-nociceptive, anti-inflammatory and antispasmodic activity [23,25,26,27,28,29,30]. Due to the diuretic, anti-inflammatory and antioxidant properties of papaloquelite, and the close relationship either these biological activities and the development of kidney disease, this plant could be an important source of compounds for the treatment of KAI. For that, the aim of this study was to evaluate the nephroprotective activity in vivo of the hydroalcoholic extract of *Porophyllum ruderale* using a thioacetamide-induced injury model, as well as, to identify the major compounds in the extract.

## 2. Results

### 2.1. Anti-Inflammatory Activity

#### 2.1.1. Cell Viability Tests

**Figure 1:**
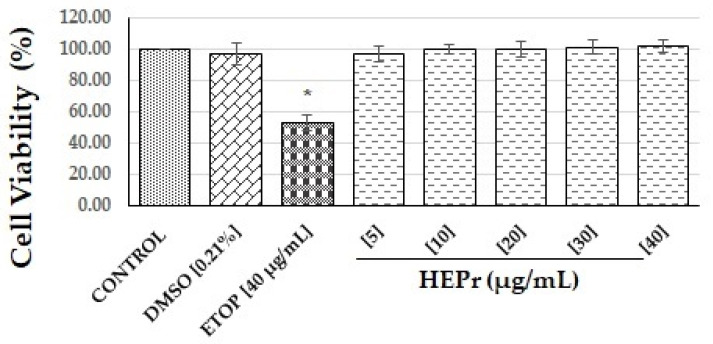
Effect of HEPr on cell viability of RAW 264.7 macrophages. Values are expressed as the mean ± SD of three independent experiments (*n* = 3). Significant difference was determined using ANOVA followed by Dunnett’s multiple comparison test. DMSO, ETOP (etoposide) and extracts compared to control group (* *p* ˂ 0.0001). Control = untreated cells, defined as 100% viability.

Anti-inflammatory activity in vitro of HEPr was determined according to Sánchez-Ramos et al. [31]. Firstly, the extract was evaluated for its effect on the viability of RAW 264.7 cells at different concentrations (5 to 40 µg/mL). The extract did not exhibit a significant reduction in the viability of macrophages compared with the control group, while the positive control (etoposide) showed a significant reduction in the cellular viability at 40 µg/mL (Figure 1).

#### 2.1.2. Inhibition of Nitric Oxide (NO) Production

**Figure 2:**
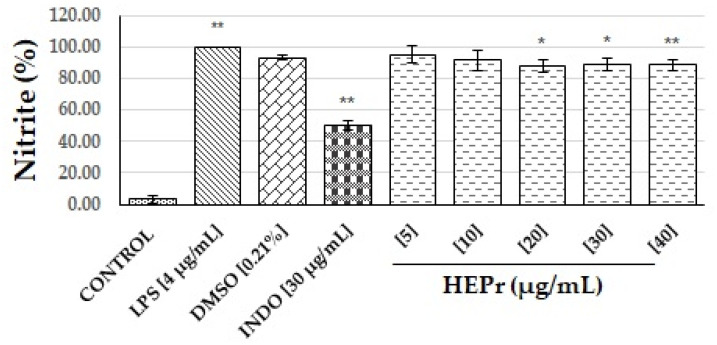
Effect of HEPr on nitric oxide (NO) production in RAW 264.7 macrophages stimulated with LPS. Values are expressed as the mean ± SD of three independent experiments (*n* = 3). The significant difference was determined using an ANOVA followed by Dunnett’s multiple comparison test. LPS compared to the control group (*p* ˂ 0.0001), and DMSO, INDO (indomethacin) and extracts compared to the LPS group (* *p* ˂ 0.001 or ** *p* ˂ 0.0001). Control = cells without stimulus.

Figure 2 shows the effect of HEPr on nitric oxide production in macrophages, compared with the negative control (cells without stimulus), the cells treated with the lipopolysaccharide (LPS) that gives the maximum inflammation, DMSO that was the vehicle and indomethacin as the reference drug. Hydroalcoholic extract of papaloquelite (HEPr) at concentrations of 20 to 40 µg/mL shows a significant difference compared with LPS; however, the extract shows a slightly anti-inflammatory effect due to it decreasing the inflammatory process by approximately 10 to 15%.

### 2.2. Antioxidant Activity

The generation of oxidative stress in the kidney is the essential mechanism of xenobiotics-induced nephrotoxicity, due to ROS damage to the cell function due to disturbing oxygen-reduction balance [32]. For this reason, HEPr antioxidant capability was determined (Table 1). Additionally, we determined the HEPr phenolic content (Table 1) because these compounds are molecules with a high potential to neutralize free radicals [33].

HEPr showed high antioxidant activity. It was able to stabilize free radicals, showing the highest effect on ABTS^+^ followed by DPPH^.^ radical (16116.03 ± 0.038 and 1502.40 ± 0.04 µmolTE/100g, respectively); it exhibited ferric reducing antioxidant power (4836.14 mgFeSO_4_/100 g). With respect to the content of phenolic compounds, HEPr showed a content of 13993.67 ± 0.016 mgGAE/100 g.

### 2.3. Acute Oral Toxicity

No animal died during the 14 days of observation after the dose of 5000 mg/kg of HEPr. The mice ate and increased their body mass normally. No signs of toxicity were observed such as difficulty in breathing, loss of appetite, or death. According to OECD standards, HEPr has an LD_50_ > 5000 mg/kg and is considered a harmless species (Table 2).

### 2.4. In Vivo Nephroprotective Activity

To evaluate the possibility that *Porophyllum ruderale* was able to prevent renal injury caused by toxic agents, we evaluated its nephroprotective activity using a thioacetamide-induced acute renal injury model in rats and determined the main biomarkers of renal injury in urine and serum of the treated rats.

**Figure 3:**
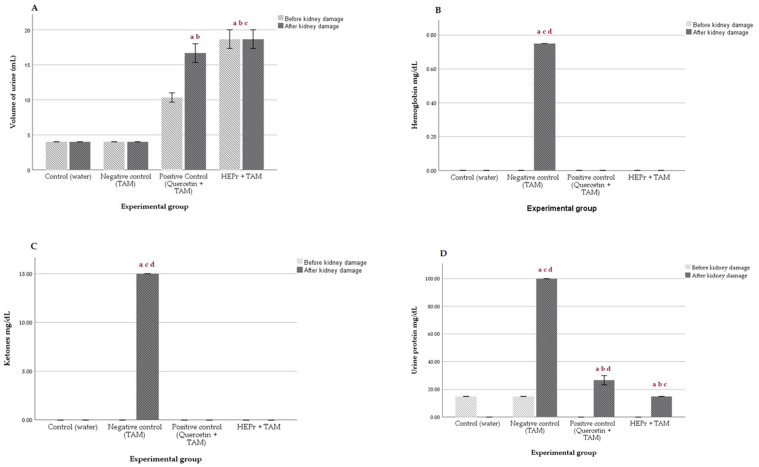

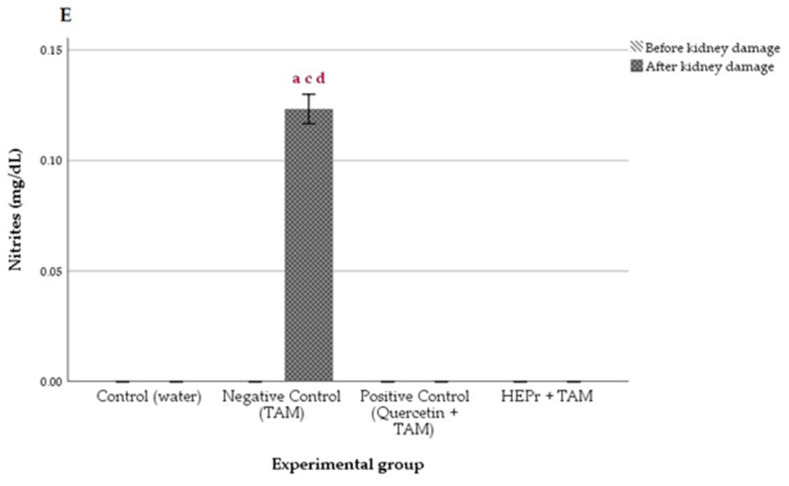
(**A**) Volume of urine, Concentrations of (**B**) Hemoglobin, (**C**) Ketones, (**D**) Proteins, (**E**) Nitrites in urine pre- and post-treatment in Wistar rats. TAM = thioacetamide. Values are expressed as the mean ± SD of urinary urine values (*n* = 5). A significant difference was determined using ANOVA followed by Dunnett’s multiple comparison test. (a) Control, (b) Negative control, (c) Quercetin, (d) HEPr + TAM group.

Figure 3A–D shows the changes in urinary biochemical markers in rats before and after renal injury induced by TAA. As we can observe previous to TAA administration, HEPr and quercetin induced an increase in the urine volume, which may be due to a diuretic effect. After TAA administration, rat groups treated with quercetin and TAA showed normal values for the rest of the biomarkers.

**Figure 4:**
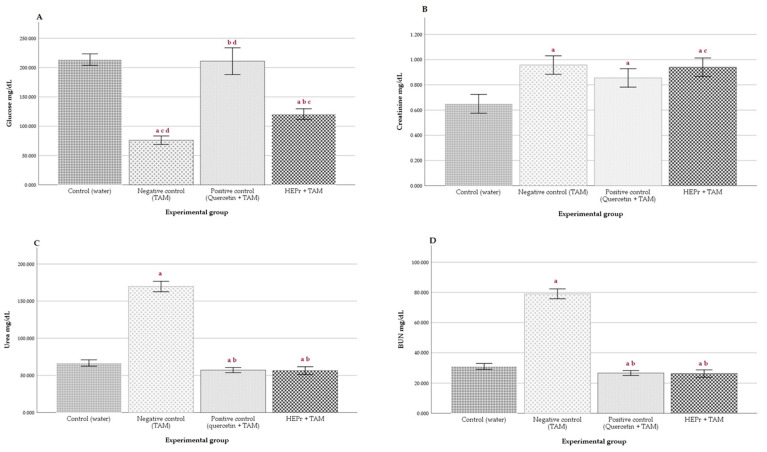
Effect of hydroalcoholic extract of papaloquelite (*Porophyllum ruderale*) (HEPr) on serum levels of (**A**) Glucose, (**B**) Creatinine, (**C**) Urea, (**D**) BUN in Wistar rats. Values are expressed as the mean ± SD of serum values of each marker (*n* = 5). Significant difference was determined using ANOVA followed by Dunnett’s multiple comparison test. (a) Control (water); (b) Negative control (TAM), (c) Positive control (quercetin), (d) HEPr + TAM group.

Concerning serum biomarkers of renal injury, we can observe that HEPr considerably decreases BUN and Urea levels without a significant difference in the control and positive control groups (Figure 4C,D). In addition, HEPr slightly decreased creatinine serum content; however, this effect was not significantly different with respect to the negative control (Figure 4B). Finally, HEPr showed a decrease in glucose content in the serum compared with quercetin and control groups, but this value was higher than that obtained in rats without treatment (Figure 4A).

### 2.5. Major Compounds

**Figure 5:**
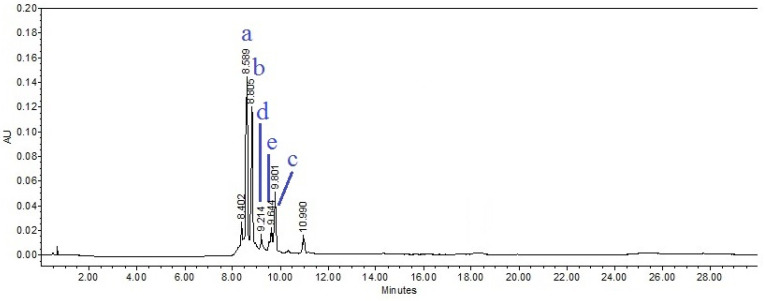
HPLC chromatogram of the hydroalcoholic extract of *Porophyllum ruderale* (HEPr) (1 mg/mL) observed at λ 330 nm. (a) 5-*O*-caffeoylquinic acid, (b) 4-*O*-caffeoylquinic acid, (c) ferulic acid, (d) quercetin-3-*O*-glucoside, (e) kaempferol-3-*O*-glucoside.

**Figure 6:**
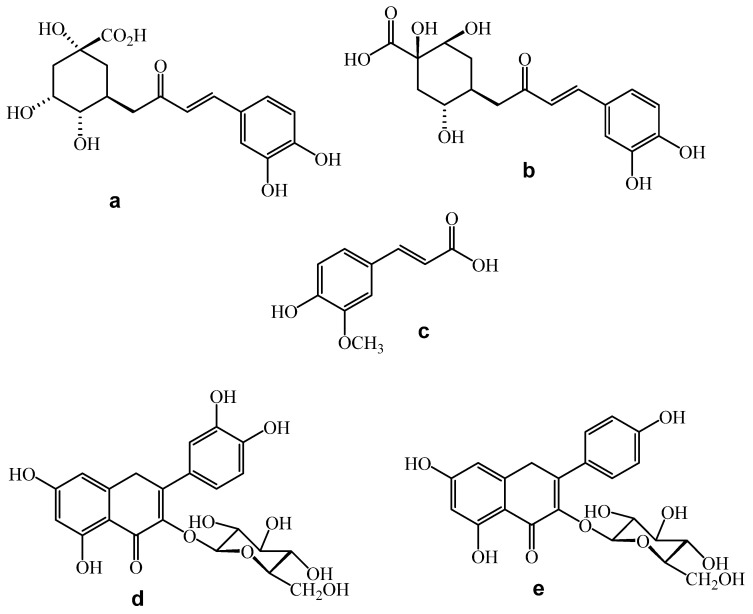
Chemical structure of compounds identified in HEPr. (**a**) 5-*O*-caffeoylquinic acid, (**b**) 4-*O*-caffeoylquinic acid, (**c**) ferulic acid, (**d**) quercetin-3-*O*-glucoside, (**e**) kaempferol-3-*O*-glucoside.

**Figure 7:**
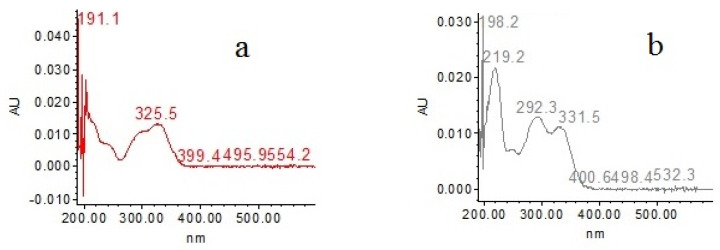
UV light spectrum of compounds in peaks at R_t_ 8.42 (**a**) and 11.058 (**b**) min.

Figure 5 shows the chromatogram obtained from HEPr at λ330 nm. The analysis of a hydroalcoholic extract of the aerial parts of *Porophyllum ruderale* by HPLC revealed that these extracts contain the phenolic acids 5-*O*-caffeoylquinic acid (Chlorogenic acid), 4-*O*-caffeoylquinic acid (cryptochologenic acid) and ferulic acid; as well as the flavanols: quercetin-3-*O*-glucoside and kaempferol-3-*O*-glucoside, as the main components (Figure 6). These phenolic acids and flavanols were identified by a direct comparison of the retention time of each peak, with the respective analytical standard and their contents shown in Table 3. Peaks of R_t_ at 8.42 and 11.058 min in the chromatogram were not identified; however, their UV light spectra showed absorption bands characteristic of caffeic acid derivatives and coumarins, respectively (Figure 7) [34,35].

## 3. Discussion

As we mentioned previously, inflammation is the main cause of acute renal failure followed by oxidative stress. For this reason, we evaluated the hydroalcoholic *P. ruderale* extract (HEPr) anti-inflammatory and antioxidant activity in vitro.

Firstly, we evaluated the toxicity of HEPr on RAW 264.7 cells (Figure 1) and we found that the extract was not toxic for cells. These results are in accordance with other studies where authors observed that methanolic extracts of other *Porophyllum* species are not cytotoxic in the mouse macrophage cell line RAW264.7 at concentrations ranging from 0.06 to 200 mg/mL [36,37]. In addition, Pawłowska et al. [30] found that an aqueous extract of aerial parts of *Porophyllum ruderale* at a concentration range of 5 to 100 µg/mL does not affect the viability of human neutrophil cells.

On the other hand, HEPr at concentrations of 20 to 40 µg/mL shows a significant difference compared with LPS (Figure 2); however, the extract shows a slight anti-inflammatory effect due to it decreasing the inflammatory process by approximately 10 to 15%. It has been observed that different plant extracts from the genus *Porophyllum* showed concentration-dependent inhibitory effects on the expression of lipopolysaccharide (LPS)-induced inflammatory marker production in inflammatory models. For instance, the essential oil of *P. ruderale* at a dose of 100 mg/kg inhibits leukocytes (37%) and mononuclear cell (43%) migration, as well as the accumulation of eosinophils (63%) induced by LPS in mice; in addition, their main monoterpenes limonene and β-myrcene were able to inhibit the migration of the same cells and production of NO, γ-interferon, and IL-4 [38]. Furthermore, Pawłowska et al. [30] observed that an aqueous extract of the aerial parts of *P. ruderale* at a concentration of 50 µg/mL decreased LPS-stimulated IL8 and TNF production by 10% in human neutrophils. Finally, a methanolic extract of *Porophyllum tagetoides* possesses compounds that help modulate NF-κB activation by decreasing LPS-induced iROS [39].

As mentioned, these activities can be attributed to the type of compounds present in this species. Several chemical studies of plants of the genus *Porophyllum* have shown that terpenes and thiophenic compounds are the main secondary metabolites [40]. These compounds can inhibit LPS-induced IκBα degradation, leading to the suppression of proinflammatory mediators such as inducible nitric oxide synthase (iNOS) and COX-2 [41].

DPPH, ABTS and FRAP assays are preliminary tests to study the antioxidant activity of plant extracts. In this investigation, HEPr was able to stabilize all radical species as well as to reduce the ferric ion to the ferrous state. Regarding DPPH activity, HEPr inhibits this radical in 63.06% and the radical inhibition of DPPH was 1502.40 ± 0.0407 µmol TE/100 g. These values are higher than that reported for an ethanolic extract of the same species (32.6 ± 1.18%, 676.24 ± 0.34 µmol ET/100 g) [40], but lesser than that obtained for wild and cultivated *P. ruderale* (4645.53 ± 36.2 and 4392.16 ± 27.0 µmol TE/100 g, respectively); however, in this case, fresh leaves were used [20].

In addition, HEPr showed good activity against ABTS and FRAP too (16116.03 ± 0.038 µmol TE/100 g and 4836.14 ± 0.072 mg FeSO_4_/100 g, respectively). It was not able to be contrasted with similar tests in the same plant genus due to a lack of publications. However, our results are higher than those presented by Khan et al. [42] for another species with high antioxidant capacity such as purple grapes (ABTS: 910 ± 0.2 µmol TE/100 g, FRAP: 2660 ± 0.9 mg FeSO_4_/100 g) and strawberries (ABTS: 1150 ± 0.4 µmol TE/100 g, FRAP: 249 ± 0.7 mg FeSO_4_/100 g).

In some plant species, the phenolic compounds present may be responsible for their antioxidant activity, due to the number of hydroxyl groups that act as free radical scavengers [43]. HEPr has a phenolic compound content similar to that reported by Kato da Silva et al. [44] for an ethanolic extract of the same plant (139.93 and 162.29 mgGAE/g, respectively). However, it was higher than the total phenol content in fresh leaves of wild and cultivated *P. ruderale* (3.91 ± 1.41 and 3.162 ± 0.28 mgGAE/g, respectively). The differences in the antioxidant activity and phenolic compounds content in *P. ruderale* aerial parts may be due to the growing conditions and edaphoclimatic characteristics of the respective geographical areas [20]. For instance, Fukalova et al. [20] obtained *P. ruderale* from the Valencian coast in Spain; while Kato da Silva et al. [44] obtained it from Campo Grande, MS, Brazil, and we obtained it from Hidalgo, México. In addition, the HEPr phenol content is higher than obtained for a crude extract of *Porophyllum tagetoides* leaves (8.54 ± 0.14 mgGAE/g) [45], and for different grape varieties (54.23 ± 0.04–58.48 ± 0.09 GAE/g) [44] (4764 ± 39–11525 ± 886 mg GA/100 g) [46,47].

Once we verified that *Porophyllum ruderale* had an antioxidant and anti-inflammatory capacity, we proceeded to evaluate its ability to protect the kidney from TAA-induced injury in rats.

The urine analysis of rats showed significant changes in the amount of urine when compared to experimental animals before and after inducing renal injury, observing an increase in the volume of urine of the animals treated with the hydroalcoholic extract of papaloquelite as shown in Figure 3A; associating it to a diuretic effect but not related to the severity of the renal injury. With respect to the other urinary parameters evaluated, urobilinogen, hemoglobin, bilirubin, ketones, glucose, protein, pH, nitrites, leukocytes, specific gravity, and the microalbumin/creatinine, were absent in animals treated with HEPr, except for the negative control group as shown in Figure 3B–E. These results for the urinary markers evaluated support the metabolic decompensation and tubular lesions associated with the renal damage of the animals not treated with the extract, as mentioned in Figure 3A–E.

On the other hand, creatinine concentration in plasma and urine is an important marker of renal function. An increase in creatinine in plasma suggests leakage from necrotic cells or upregulates creatine biosynthesis. Creatinine is synthesized and metabolized in the liver, but its precursor guanidinoacetate is formed in the kidney, transported through the blood, and undergoes methylation in the liver to form creatine which enters the blood for use in peripheral tissues [48]. Furthermore, because kidney damage progresses, nitrogen products accumulate in proportion to the loss of kidney function. The blood marker blood urea nitrogen (BUN) measures the amount of accumulated urea that is not efficiently excreted in the urine, making it an important marker of kidney damage [49,50]. As well as urea, which is synthesized in the liver as an end product of protein catabolism and subsequently eliminated in the kidney via the urine, its accumulation can exert toxic effects, leading to cell death by induction of apoptosis [51,52].

In our experiment, the negative control group treated with TAA exhibited an increase in serum creatinine and urea concentration, 30% and 166%, respectively. These values are according to other authors who found that a single dose of 150 mg/kg of thioacetamide administered orally to rats produced acute renal injury and altered kidney function [53]. They observed an increase in creatinine and urea serum content in TAA-treatment rats (33 and 168%, respectively) 24 h after toxic administration. Furthermore, vacuolar degeneration in kidney tubules was observed. In addition, rats in the negative control group showed an increase in BUN of 169.5% compared to the control group, while for the groups administered with papalo and quercetin, the values decreased by 14.2 and 11.12%, respectively (Figure 4D). Alterations in these biological markers are evidence of renal injury in the rats.

As we can observe in Figure 4A–D; the values of the markers in serum were maintained in normal parameters for animals treated with HEPr, showing serum glucose levels of 114.62 ± 7.5 mg/dL, creatinine 0.85 ± 0.16 mg/dL; urea 76.34 ± 16.29 mg/dL and urea nitrogen 35.57 ± 7.59 mg/dL. An opposite behavior was observed in untreated animals, where the values of the markers of renal damage were increased, being important markers of acute damage besides being associated with alterations in the renal homeostasis of nutrients [54]. Data found in this study are similar to those observed for olive and juniper and flaxseed oils in rats with kidney damage instated with TAA, where serum creatinine (+38.5% and +34.5%, respectively) and BUN (+26.3 and +30.1%, respectively) levels were elevated in TAA-treated mice compared to control group [55,56]. In addition, these parameters were not statistically changed in rats treated with flaxseed oil plus TAA compared to control rats [56].

Moreover, the results of this study are similar to those presented by Cengiz [11]. This author induced acute kidney injury in rats with TAA too and observed that the group treated only with TAA had elevated BUN values (18.42 ± 0.71 mg/dL) compared to the group treated with 100 mg/kg of *Silybum marianum* (L.) Gaertn (Silymarin) and 50 mg/kg TAA, which showed similar values to those reported for the control group (15.89 ± 1.32 and 15.19 ± 1.73 mg/dL, respectively).

In another study, acute kidney disease in rats was induced with TAA (single dose of 300 mg/kg) and rats were treated for 14 days with an alcoholic extract of *Allium porrum* and *Bauhinia variegata* leaves. Authors observed that creatinine and urea values were higher in the group with TAA (1.53 ± 0.08 and 77.34 ± 3.16 mg/dL, respectively); while in the groups treated with the plant species, these values decreased (1.15 ± 0.02 and 41.86 ± 1.07 mg/dL, respectively) showing a nephroprotective effect [57].

In comparison with the results of a 12-week chronic kidney disease trial evaluating the effect of olive and juniper leaf extracts on thioacetamide (TAA)-induced nephrotoxicity in male rats, it was observed that after 12 weeks, serum creatinine (+38.5%) and BUN (+26.3%) levels were elevated in TAA-treated mice compared to control mice (25.50 ± 1.64; 5.67 ± 0.72 µmol/L respectively). However, unlike what was found in our study, there were no significant changes in serum creatinine (24.50 ± 2.88 µmol/L) and BUN (5.36 ± 1.04µmol/L) levels in mice treated with TAA plus olive and juniper leaf extract [52].

On the other hand, the nephroprotective effect of *Vitex negundo* (VN) ethanolic extract was evaluated for 12 weeks in a TAA-induced chronic kidney disease model [58]. The TAA group showed a significant increase in blood urea and serum creatinine levels (35.66 ± 7.3 and 4.46 ± 0.45 mmol/L, respectively) compared to the normal control group; while the increase in these parameters was prevented by simultaneous treatment of the animals with 100 mg/kg of VN (Creatinine: 6.28 ± 0.59 mol/L; Urea: 35.66 ± 7.3 mmol/L) and 300 mg/kg of VN (Creatinine: 5.73 ± 0.973 mol/L; Urea: 29.33 ± 3.4 mmol/L), which resulted in almost normalized levels of these parameters.

Regarding the chemical composition of *Porophyllum ruderale*, we identify 5-*O*-caffeoylquinic acid (Chlorogenic acid) and 4-*O*-caffeoylquinic acid (cryptochologenic acid) as the most abundant compounds followed by ferulic acid, quercetin-3-*O*-glucoside, and kaempferol-3-*O*-glucoside (Figure 5). Recently, 25 phenolic compounds were identified in the acetone:methanol:water (3:1:1 *v/v/v*) extract of the aerial parts of *P. ruderalle* cultivated in Warsaw, Poland. These compounds were identified by UHPLC-DAD-MS as thirteen caffeic acid derivatives, ten flavonoids, one p-coumaric acid derivative and one unknown compound. The most abundant compounds were 2-*O*-caffeoyl-2C-methyl-D-erythronic acid, 5-*O*-caffeoylquinic acid, quercetin-3-*O*-β-D-glucuronide and 3-*O*-caffeoyl-2C-methyl-D-erythronic acid (152.59, 143.77, 65.54 and 54.70 mg/g extract, respectively). Additionally, quercetin 3-*O*-β-D-glucopyranoside, kaempferol 3-*O*-D-glucopyranoside, 4-*O*-caffeoylquinic acid and 3-*O*-caffeoyl quinic acid were identified too [30]. In another study performed on *P*. *ruderale* from Brazil, authors found chlorogenic acid and quercetin-3-*O*-glucoside as major compounds [59]. Chlorogenic acid was the main component of *P. ruderale* from Spain, followed by *p*-coumaric acid, quercetin, rutin, kaempferol, luteolin, caffeic acid, apigenin and gallic acid [20]. Authors suggest that differences in chemical composition may be due to the growing conditions and edaphoclimatic characteristics of the respective geographical areas [20].

*Porophyllum ruderale* is a great source of phenolic compounds with important anti-inflammatory and antioxidant activities that can contribute to its nephroprotective effect. For example, chlorogenic acid has antioxidant activity and protects cells from oxidative stress [60]; in addition, it is able to inhibit nitric oxide production by macrophages and suppress T cell proliferation, decreasing inflammatory processes [61]. While, ferulic acid has a potent antioxidant activity mediated mainly by its binding to free radicals to donate hydrogen molecules, as well as inhibit superoxide anion [62,63,64,65,66]. Additionally, it decreases the levels of different inflammatory mediators such as prostaglandin E2 and TNFα, as well as the expression of the enzyme NOS (nitric oxide synthase) [67].

Furthermore, quercetin glycoside also has important antioxidant activity, acting as a protector against reactive oxygen species by neutralizing free radicals such as superoxide anions, nitric oxide and peroxynitrite, as well as increasing the production of endogenous antioxidants [68]. In addition, this compound can decrease the inflammatory mediators produced by macrophages [69]. Finally, in some studies, it has been observed that quercetin glycoside shows protection against nephrotoxicity by reducing renal toxicity from exposure to cisplatin and cadmium [70,71,72].

On the other hand, it has been reported that coumarins have antioxidant activity related to their ability to inhibit lipid peroxidation and scavenge reactive species, e.g., hydroxyl and superoxide radicals [73]. Several coumarins have also shown beneficial biochemical profiles in relation to pathophysiological processes that depend on reactive oxygen species [74]. In addition, several coumarins isolated from plants have been identified as having significant anti-inflammatory and/or analgesic activities [75].

Finally, it is known that renal damage is associated with pro-oxidant mechanisms that alter the structure and function of renal glomeruli, activating apoptotic pathways and glomerular inflammatory lesions caused by mediators such as cytokines and chemokines, which provoke leukocyte activation, ROS production, and increased glomerular damage [76,77]. These data indicate that ROS activate the secretion of inflammatory molecules and these, in turn, exert effects mediated by ROS, originating a cycle that perpetuates the inflammatory response, so that the nephroprotective activity presented by HEPr in this study is due to the presence of compounds with antioxidant and anti-inflammatory properties.

The analysis of the data presented in this study provides a basis for a potential therapeutic intervention in renal oxidative damage in humans, which could be used as an adjuvant treatment to prevent, mitigate the progression of, or attenuate the renal damage caused by oxidative stress.

## 4. Materials and Methods

### 4.1. Plant Material

The aerial parts of papaloquelite (*Porophyllum ruderale*) were collected in Santa Ana Hueytlalpan, Tulancingo, Hidalgo, México in May 2019. A specimen was deposited at the herbarium of the Faculty of Higher Education Iztacala of the National Autonomous University of Mexico. The species was identified by MSc. Ma. Edith López Villafranco with the code number 3350 IZTA. The rest of the plant material was dried in the dark at room temperature, grounded and stored in hermetic bags, keeping it refrigerated until use.

### 4.2. Preparation of Hydroalcoholic Extract (HEPr)

The dried *P. ruderale* aerial parts (3 kg) were macerated with an aqueous methanol solution (70%, 1:2 ratio *w*/*v*) at room temperature for 24 h, this operation was realized three times. After, the extract was filtered, and the filtrate was distilled under reduced pressure on a rotary evaporator (Büchi, R-215) to remove the solvent. The solid extract was stored at −20 °C until biological testing.

### 4.3. In Vitro and In Vivo Test

Firstly, we determined the anti-inflammatory and antioxidant activity of HEPr in vitro and after that, we evaluated its toxicity and nephroprotective activity in vivo.

#### 4.3.1. Anti-Inflammatory Activity

The in vitro anti-inflammatory activity of HEPr was determined according to Sánchez-Ramos et al. [31].

##### Cell Culture

The murine macrophage cell line RAW 264.7 (Tib-71^TM^ from ATCC) was maintained in DMEM/F12 medium supplemented with 10% heat-inactivated fetal bovine serum without antibiotics. Cells were cultured at 37 °C in a humidified atmosphere containing 5% CO_2_ for 24 h.

##### Cell Viability by MTS Assay

To determine the cell viability, RAW 264.7 cells were seeded in a 96-well plate (10,000 cells/well) with 0.1 mL of culture medium and incubated for 24 h. A stock solution (3 mg/mL) of extract and positive control (etoposide) was prepared using DMSO as a solvent, and later dilution with culture medium was performed to obtain the working solutions, which allowed applying the samples into the wells of the cell culture plate; the maximum final concentration of DMSO was 0.21%. In this way, the cells were treated with the extracts at various concentrations (5–40 µg/mL) or vehicle (DMSO, 0.21%, *v/v*) or etoposide (40 µg/mL) that served as a positive control and was incubated for 22 h. After 22 h, cell viability was determined by the MTS assay. Briefly, 20 µL of MTS solution (Promega) was added to each well and incubated for another 2 h. Optical density was measured at λ 490 nm in an ELISA plate reader.

##### Treatment of Macrophages with Lipopolysaccharide (LPS)

RAW 264.7 cells were seeded in a 96-well plate (20,000 cells/well) with 0.2 mL of culture medium and incubated for 24 h. Extract and indomethacin were dissolved in DMSO and then diluted with culture medium in the same way as in the cell viability assay. Subsequently, the cells were treated with the extract at concentrations that do not affect cell viability or vehicle (DMSO, 0.21%, *v/v*) or indomethacin (30 µg/mL) that served as a positive control and incubated for 1 h. Next, the LPS pro-inflammatory stimulus was applied at 4 µg/mL to the wells that were treated with extracts, vehicle and indomethacin, leaving wells with cells that were only treated with LPS (100% stimulus control) and wells with cells without any treatment (negative control), and incubated at 37 °C for 20 h. Finally, cell-free supernatants were collected and used fresh for NO quantification.

##### Determination of NO Concentration

For the determination of NO, the nitrite-stable final product of nitric oxide (NO) was used as an indicator of its production in cell supernatants, and it was measured according to the Griess reaction. Briefly, in a fresh 96-well plate, 50 µL of each supernatant was mixed with 100 µL of Griess reagent [50 µL of 1% sulfanilamide and 50 µL of 0.1% N-(1-Naphthyl) ethylenediamine dihydrochloride in acid solution; 2.5% phosphoric] and incubated for 10 min at room temperature. The optical density was measured at 540 nm (OD_540_) in an ELISA plate reader and the nitrite concentration in the samples was calculated by comparison with the OD_540_ of a standard curve of NaNO_2_ prepared in a fresh culture medium.

#### 4.3.2. In Vitro Antioxidant Activity Assays

DPPH (1,1-diphenyl-2-picrylhydrazyl), ABTS (2,2′-azino-bis(3-ethylbenzothiazolin)-6-sulfonic acid) and FRAP (Ferric Reducing Potential) techniques were used to evaluate antioxidant activity. Additionally, the total phenols content was determined. All experiments were performed in triplicate using a BioTek 146,583 PowerWave HT microplate spectrophotometer (Agilent; Santa Clara, CA, USA)and Gen 5 version 2.09 software (Agilent; Santa Clara, CA, USA).

##### DPPH Radical Scavenging Assay

To quantify the free radical scavenging capacity of the extract HEPr, the degree of decolorization caused by their components in an ethanolic solution of DPPH was determined by the [32] method with some modifications.

One hundred micrograms of HEPr were dissolved in 10 mL of methanolic solution (70%); 100 μL of the solution was mixed with 500 μL of 0.1 mM DPPH solution in ethanol. The plates were incubated in the dark at room temperature for 60 min. Finally, the optical density was measured at λ 517 nm in a microplate spectrophotometer, using ethanol as a reference blank. Trolox was used as a reference and results were expressed in μmol Trolox equivalents per gram of extract (μmol TE/g) [33].

##### ABTS Radical Scavenging Assay

One hundred micrograms of HEPr were dissolved in 10 mL of methanolic solution (70%). The radical was generated by the reaction of a 7 mM solution of ABTS in deionized water with 2.45 mM K_2_S_2_O_8_ (1:1 *v/v*). The solution was held in darkness at room temperature for at least 16 h to obtain stable absorbance values at λ 734 nm. Subsequently, 20 µL of the extract solution was added to 980 µL of the ABTS radical, vortexed and allowed to stand for 7 min. Then, 200 µL of the vial was poured into four different wells of a microplate and the absorbance was read at λ 754 nm using distilled water as a reference blank. The results were expressed in μmol Trolox equivalents per gram of extract (μmol TE/g) [78].

##### Ferric Reducing Antioxidant Power (FRAP) Assay

An amount of 100 mg of HEPr was dissolved in 10 mL of methanolic solution (70%); 30 µL of extract solution was mixed with 90 µL of distilled water and 900 µL of FRAP reagent. FRAP reagent contained 2.5 mL of 10 mM TPTZ solution in 40 mM HCl, 2.5 mL of 20 mM FeCl_3_ and 25 mL of 300 mM acetate buffer (pH 3.6). Solutions were vortexed and incubated in a water bath at 37 °C for 10 min. Then, 200 µL of the vial was poured at room temperature into four different wells of a microplate, the absorbance was read at λ 593 nm using distilled water as a reference blank. The results were expressed as mg FeSO_4_/g [79,80].

##### Total Phenolic Content

The determination of phenol content was carried out using the Folin and Ciocalteu method with some modifications, using 100 µL of the dilution 100:10 of HEPr, 500 µL of Folin–Ciocalteau reagent (10% *v/v*) and 400 µL of sodium carbonate (7.5% *w/v*). The sample was vortexed and left to stand for 30 min in the absence of light. After this time, 200 µL of each vial was poured into four wells of a microplate to finally obtain readings at λ 760 nm, the results were expressed in mg of gallic acid per 100 g of the extract [81].

#### 4.3.3. In vivo Acute Oral Toxicity

The acute oral toxicity test was determined based on the methods described in the OECD Guideline for Testing of Chemical “Acute Oral Toxicity Acute Toxic Class Method” No. 423 Adopted on 20 December 2001 [82]. This test is based on the use of a dose progression factor from 5 to 2000 mg/kg, while for extracts for which no toxic effect is known, it is recommended to start with the limit test (5000 mg/kg).

For this study, five male mice of the CD1 strain of 39 g, were maintained under standard 12-h light/dark cycle conditions at 22 °C and 45% relative humidity control. They were provided with food and water *ad libitum*.

Prior to each experiment, the animals were left in food deprivation for 12 h and then were administered a single dose of 5000 mg/kg of the HEPr intragastric and vehicle to the control, since no data on the toxicity of the species to be evaluated were found. The administration of the extract was performed as follows: mice 1, 2, 3 and 4 received 195 mg of HEPr diluted in 1 mL of water, while mouse 5 received 1 mL of water.

The animals were kept under post-administration observation for 14 days, with special attention during the first 4 h. Body weight was recorded every third day and toxic signs were recorded daily, including piloerection, difficulty in breathing, loss of appetite and death. At the end of the observation period, the animals were euthanized by cervical dislocation.

#### 4.3.4. In Vivo Nephroprotective Activity

All procedures described in this project were carried out in accordance with the Mexican Official Standard NOM-062-ZOO-1999: Technical specifications for the production, care and use of laboratory animals; in addition to being approved by the Ethics Committee for the care and use of laboratory animals of the Autonomous University of the State of Hidalgo, with the following approval number: CICUAL/003/2021.

A total of twenty male albino Wistar rats weighing 250–300 g were used for the present study. The animals were housed in metabolic boxes, given standard rat chow and drinking water, and maintained under a controlled temperature (22 °C), with a 12 h light/12 h dark cycle; prior to each experiment, the animals were left in food deprivation for 12 h. The animals were haphazardly categorized into four groups, each containing five rats, as follows:

Group 1 (Control group): 0.5 mL of water *i.g*.

Group 2 (Negative control): 100 mg/kg of TAA dissolved in saline solution *i.p.*

Group 3 (Positive control): pretreatment for 4 days with 50 mg/kg quercetin *i.g.* At day 5, 50 mg/kg quercetin *i.g.* and 100 mg/kg TAA *i.p.* were administered.

Group 4: pretreatment for 4 days with 100 mg/kg HEPr i.g. At day 5, 100 mg/kg of the extract was administered intragastric together with 100 mg/kg of TAA *i.p.*

The experiment began with the administration of HEPr and quercetin, diluted in 1 mL of distilled water to the corresponding groups. Four days after the beginning of the treatment, a single dose of thioacetamide (TAA) dissolved in 1 mL of NaCl (0.9%) was administered intraperitoneally to groups 2, 3 and 4 to produce acute renal injury. Twenty-four hours after the administration of TAA, the groups were euthanized by exsanguination by portal vein puncture in animals previously sedated with 1 mL of veterinary ketamine/xylazine.

##### Biochemical Assays

Tests were performed on pre- and post-treatment urine samples, using a portable digital urine analyzer (SONOMEDIC, Cd. Obregón, Sonora, México) and test strips (Brand: Mission; Model: Acon), for the evaluation of the following parameters: urobilinogen, blood, bilirubin, ketones, glucose, protein, pH, nitrites, leukocytes, specific gravity and the microalbumin/creatinine ratio.

Subsequently, the blood samples taken were analyzed for the quantitative determination of biochemical parameters markers of renal damage in serum, using SPINREACT (Girona, Spain) kits for each of the parameters analyzed: glucose (SPINREACT 41010), creatinine (SPINREACT 1001111), protein (SPINREACT 1001291), urea (SPINREACT 1001326) and urea nitrogen (SPINREACT 1001323).

### 4.4. Identification of Major Compounds of HEPr

EHPr was analyzed by HPLC in order to identify their chemical composition.

Chromatographic analysis was performed according to [83]. Briefly, a Waters 2695 separation module system equipped with a Waters 996 photodiode array detector and Empower Pro software (Waters Corporation, USA) was used. Chemical separation was achieved using a Discovery C18 column (4.6 × 250 mm i.d., 5-μm particle size) (Sigma-Aldrich, Bellefonte, PA, USA). Two gradient elution methods were used. For both methods, the mobile phase consisted of a 0.5% trifluoroacetic acid aqueous solution (solvent A) and acetonitrile (solvent B). The gradient system of the first method was as follows: 0–1 min, 0% B; 2–3 min, 5% B; 4–20 min, 30% B; 21–23 min, 50% B; 24–25 min, 80% B; 26–27 100% B and 28–30 min, 0% B. The flow rate was maintained at 0.9 mL/min and the sample injection volume was 10 μL of sample diluted in methanol. 5-*O*-caffeoylquinic acid (chlorogenic acid), 4-*O*-caffeoylquinic acid (cryptochologenic acid), ferulic acid, quercetin3-*O*-glucoside and kaempferol-3-*O*-glucoside analytical standards were purchased from Sigma-Aldrich^®^. Content of compounds in the extract was determined according to areas under the curve.

### 4.5. Statistical Analysis

The results shown were obtained from at least three independent experiments and are presented as the means ± standard deviation. Statistical analysis was performed by one-way analysis of variance (ANOVA), followed by Dunnett’s multiple comparisons test. All statistical analyses were performed using IBM SPSS, Statistics for Windows, Version 26.0. Armonk, NY: IBM Corp. The *p* < 0.05 level of probability was used as the criteria of significance.

## 5. Conclusions

This work showed that *Porophyllum ruderale* has a nephroprotective effect against AKI induced by thioacetamide. This pharmacological property may be due to the presence of hydroxycinnamic acids and flavanol glycosides in the plant, which have antioxidant and anti-inflammatory activities. This study becomes a very important step to give added value and promote the consumption of this species that is endemic to Mexico and whose consumption has been a very ancient practice; in pre-Hispanic times, the Aztecs used it in traditional medicine and as a vegetable to accompany food; however, despite its nutritional and pharmacological properties, it is little valued and its use in the diet has been displaced by other vegetables decreasing its purchase in traditional markets.

Furthermore, as mentioned throughout the article, the phytochemical content of the studied extract and its biological activities make it a candidate as a functional ingredient in the elaboration of widely used products. On the other hand, future studies are needed that can help identify products in which the biological value of this species can be applied, positively influencing health by increasing the intake of the constituents found in this extract.

## Figures and Tables

**Table 1:** Total phenol content and antioxidant capacity of hydroalcoholic extract of papaloquelite (*Porophyllum ruderale*).

Sample	Total Phenolics (mgGAE/100 g)	ABTS (µmol TE/100 g)	% Inhibition	DPPH (µmol TE/100 g)	% Inhibition	FRAP (mg FeSO_4_/100 g)	% Inhibition
HEPr	13993.67 ± 0.016	16116.03 ± 0.038	32.96 ± 2.496%	1502.40 ± 0.0407	63.06 ± 1.733%	4836.14 ± 0.072	69.04 ± 1.958%

**Table 2:** LD_50_ of the hydroalcoholic extract of papaloquelite (*Porophyllum ruderale*).

Phase	Intragastric Dose (mg/kg)
Phase I	5000
Mortality	0/5
LD_50_	>5000

**Table 3:** Content of compounds identified by HPLC in the hydroalcoholic extract of *Porophyllum ruderale* (HEPr).

Compound	mg/g Extract
5-*O*-caffeoylquinic acid	310.82
4-*O*-caffeoylquinic acid	340.39
Quercetin-3-*O*-glucoside	24.06
Kaempferol-3-*O*-glucoside	23.00
Ferulic acid	137.87

## Data Availability

Not applicable.
